# Effect of meteorological factors on clinical malaria risk among children: an assessment using village-based meteorological stations and community-based parasitological survey

**DOI:** 10.1186/1471-2458-7-101

**Published:** 2007-06-08

**Authors:** Yazoumé Yé, Valérie R Louis, Séraphin Simboro, Rainer Sauerborn

**Affiliations:** 1 African Population and Health Research Centre (APHRC), Shelter Afrique Centre, 2nd floor, Longonot Road, Upper Hill, P.O Box 10787-00100 GPO, Nairobi Kenya; 2 Department of Tropical Hygiene and Public Health, University of Heidelberg, Medical School, Im Neuenheimer Feld 324, D-69120 Heidelberg, Germany; 3 Nouna Health Research Centre, BP 02 Nouna, Burkina Faso

## Abstract

**Background:**

Temperature, rainfall and humidity have been widely associated with the dynamics of malaria vector population and, therefore, with spread of the disease. However, at the local scale, there is a lack of a systematic quantification of the effect of these factors on malaria transmission. Further, most attempts to quantify this effect are based on proxy meteorological data acquired from satellites or interpolated from a different scale. This has led to controversies about the contribution of climate change to malaria transmission risk among others. Our study addresses the original question of relating meteorological factors measured at the local scale with malaria infection, using data collected at the same time and scale.

**Methods:**

676 children (6–59 months) were selected randomly from three ecologically different sites (urban and rural). During weekly home visits between December 1, 2003, and November 30, 2004, fieldworkers tested children with fever for clinical malaria. They also collected data on possible confounders monthly. Digital meteorological stations measured ambient temperature, humidity, and rainfall in each site. Logistic regression was used to estimate the risk of clinical malaria given the previous month's meteorological conditions.

**Results:**

The overall incidence of clinical malaria over the study period was 1.07 episodes per child. Meteorological factors were associated with clinical malaria with mean temperature having the largest effect.

**Conclusion:**

Temperature was the best predictor for clinical malaria among children under five. A systematic measurement of local temperature through ground stations and integration of such data in the routine health information system could support assessment of malaria transmission risk at the district level for well-targeted control efforts.

## Background

Meteorological factors are important drivers of malaria transmission. Temperature, rainfall and humidity have been associated with the dynamics of malaria vector population and, therefore; with spread of the disease. Ambient temperature plays a major role in the life cycle of the malaria vector. The development of the parasite within the mosquito (sporogonic cycle) is dependent on temperature. The sporogonic cycle takes about 9 to 10 days at temperatures of 28°C, but stops at temperatures below 16°C [1-3]. The daily survival of the vector is dependent on temperature as well. At temperatures between 16°C and 36°C, the daily survival is about 90%. This survival drops rapidly at temperature above 36°C. The highest proportion of vectors surviving the incubation period is observed at temperatures between 28° and 32°C [4]. The gonotrophic cycle, which is the time between two blood meals of the vector, is short at higher temperatures because the digestion speed increases [5]. Therefore, higher temperatures result in more frequent vector-host contact.

Several field studies have reported the impact of ambient temperature on malaria outcomes. [6-10]. In South Africa, Craig and colleagues [8] identified a significant correlation between temperature and the number of malaria cases. Mean maximum daily temperatures from January to October of the preceding season were positively associated with the incidence of clinical cases of malaria. In Ethiopia, minimum temperature was associated with malaria in a cold district (minimum temperature below 12°C); while in a hot district (minimum temperature above 12°C) the effect was not significant [9].

Rainfall provides breeding sites for mosquitoes to lay their eggs, and ensures a suitable relative humidity of at least 50 to 60% to prolong mosquito survival. Relative humidity below 60% shortens the life span of the mosquitoes. The onset of the rainy season was associated with an increase in vector abundance [11,12]. The 1958 malaria epidemic in Ethiopia was associated with unusual high amounts of rain. Similarly, in Nairobi, outbreaks of malaria occurred in 1940 after heavy rains [13]. In the Ugandan highlands, rainfall anomalies (differences from the mean) because of El Nino were positively correlated with vector density one month later, and this may have started the resulting epidemic [14].

Despite consistently reported evidence that meteorological factors affect malaria transmission, conflicting opinions persist about their relative importance. Shanks and colleagues [15] could not find a link between ambient temperature and malaria admissions over a 30-year period in Kenya. Similarly, Hay and colleagues [16] attributed the increase in malaria morbidity at four sites in East Africa to drug resistance, rather than changes in temperature. Patz argued that this discrepancy might arise from methodological issues. In these studies, climate data were obtained by interpolating broad-scale grid regional climate data, based on sparse historical meteorological station data, which are unsuitable for individual village sites [17]. This was described as an absence of evidence, rather than evidence of absence of a climate effect. To assess climate factors and infectious disease outcomes, Kovats and colleagues recommended that enough measurements on each aspect should be performed for a specific study region [18].

The purpose of the current study was to estimate the effects of temperature, rainfall, and relative humidity on the incidence of clinical malaria, by examining data on these meteorological factors and laboratory-confirmed cases of clinical malaria. A binary-response logistic regression model with fractional polynomial (FP) transformations was used to assess these effects. The FP approach allowed us to explore better the nature of the relationships between these meteorological parameters and the risk of clinical malaria. This is because presumed linearity of these relationships is not justified [1-3].

## Methods

### Study site

**Figure 1:**
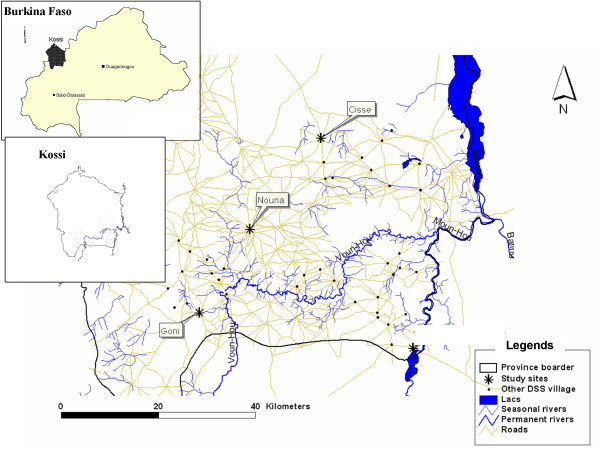
Study site location.

The study was conducted in the town of Nouna and the villages of Cissé and Goni. These three sites are part of the Nouna Demographic Surveillance Systems (DSS) area [19], which is located in Kossi province in the north-western part of Burkina Faso (Figure 1).

### Study population

Children were selected at each site by cluster sampling of households, using a sampling frame produced from the DSS database. The sample size was set to detect inter-site differences in clinical malaria incidence of at least 10%, with 80% power and 95% confidence, and accounting for 15% loss to follow up. Children were involved after getting informed consent from their parents. In total, 676 children (Cissé: 171, Goni: 240 and Nouna: 265), aged 6 to 59 months, took part in the study. The Nouna ethical committee approved the study.

### Clinical malaria detection

Three trained interviewers, one based at each site, visited each child at home every week. At each visit, they measured the child's axillary temperature and collected a blood film (by finger prick) from any child who was febrile (axillary temperature > = 37.5 C). In addition, interviewers collected information on bed net use and housing conditions. Blood films were read in the Laboratory of the Nouna Health Research Centre. Details on the clinical malaria detection procedure are provided elsewhere [20]. The outcome measure was clinical malaria episode, defined as being febrile with parasiteamia.

For purposes of this study, incidence was defined as the number of clinical malaria episodes detected per 1000 units of person time bearing in mind that some illness episodes which occurred between visits may have been missed.

For ethical reasons, children with fever were treated presumptively with cholorquine (CQ) according to the then national treatment guidelines for malaria. When fever persisted for two days or other symptoms surfaced, the interviewers immediately referred the child to the nearest health centre. The project covered all related costs.

### Meteorological data

**Figure 2:**
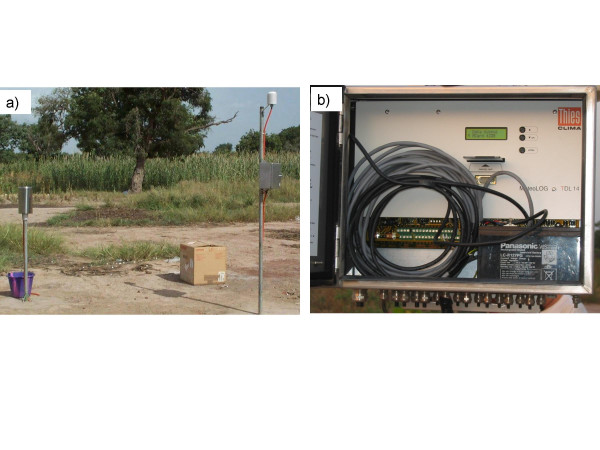
Meteorological station (a) and datalogger (b) in one of the study sites.

Rainfall, temperature, and humidity were measured on the ground using meteorological units installed at each of the three sites. Each meteorological unit consisted of a Digital Datalogger (THIES Datalogger, MeteoLOG TDL 14), to which three sensors (temperature, humidity, and rainfall) were connected (Figure 2). Following standard meteorological conventions, the temperature and humidity sensors were set at a height of 2.5 metres and the rainfall sensor 1.5 metres above the ground. The Dataloggers were set for 10-second measurement cycles and 10-minute recording cycles. Every month, a meteorological supervisor visited each site, downloaded the data from the Datalogger into a memory card, and transferred them into a meteorological database. Daily minimum, mean, and maximum temperatures, relative humidity, and total rainfall were then calculated.

### Statistical Analysis

A conventional binary outcome logistic regression model was used to assess the effects of mean, minimum, and maximum temperatures (T°C), relative humidity (RH), and amount of rainfall (Pmm) of the previous month on clinical malaria rates among study participants. The interaction terms (T°C*RH', 'T°C* Pmm' and 'RH*Pmm') were included in the model to control for the high correlation of the three individual variables. Other covariates included in the model were site of the study; sex; age; use of bed net; type of house; presence of a well; presence of a farm (presence of any farming; which included cereal and vegetable farms); presence of animals; and presence of a mosquito breeding site within a 30-metre radius of a participant's house. As the temperature, rainfall and relative humidity are continuous variables and their relationships with clinical malaria might not be linear, multivariate FP procedures [21,22] were used to determine the best-fitting relationship they had with clinical malaria. The fitting procedure involves transforming a continuous variable, using a class of eight possible functions to identify the one that provides the best fit. These functions are *H*_1_(*X*) = *X*^*p*^, where *p *takes eight possible values: -2, -1, -0.5, 0, 0.5, 1, 2, 3. The linear model is represented by *X*^1^, and *X*^0 ^represents the logarithm of *X*. The transformation can be either first or second-degree [21,22].

For the simplest model, the one with one continuous variable (e.g. temperature) and one binary variable (e.g. bed net use, Yes or No), the logistic regression model using the first-degree FP is

log*it*(*π*_*ib*_) = *α *+ *β*_1_H_1_(*X*_*i*_) + *γW*_*b*_

... Where π_*ib *_is the predicted probability of a child testing positive for clinical malaria at a temperature *i*, and for bed net use *b *(yes or no); *H*_1 _(X) is the functional form to which the co-variable X is transformed; *β*_1 _its coefficient; and γ is the coefficient of the co-variable; *W*_*b*_, the use of a bed net. The rate ratio for the first-degree PF transformation is given by the formula:

*RR *= exp (β * (H_1_(*X*_1_) - *H*_1_(*X *_0_)))

Second-degree transformation uses a combination of two powers from the list of eight. In total, 36 combinations are possible. This is calculated as follows:Ckn=n!k!(n−k)!
 MathType@MTEF@5@5@+=feaafiart1ev1aaatCvAUfKttLearuWrP9MDH5MBPbIqV92AaeXatLxBI9gBaebbnrfifHhDYfgasaacH8akY=wiFfYdH8Gipec8Eeeu0xXdbba9frFj0=OqFfea0dXdd9vqai=hGuQ8kuc9pgc9s8qqaq=dirpe0xb9q8qiLsFr0=vr0=vr0dc8meaabaqaciaacaGaaeqabaqabeGadaaakeaacqWGdbWqdaqhaaWcbaGaem4AaSgabaGaemOBa4gaaOGaeyypa0ZaaSaaaeaacqWGUbGBcqGGHaqiaeaacqWGRbWAcqGGHaqicqGGOaakcqWGUbGBcqGHsislcqWGRbWAcqGGPaqkcqGGHaqiaaaaaa@3C51@, where *n *is the number of possible powers (8), and *k *the number of powers in each combination (2); thenC28=8!2!(8−2)!=28
 MathType@MTEF@5@5@+=feaafiart1ev1aaatCvAUfKttLearuWrP9MDH5MBPbIqV92AaeXatLxBI9gBaebbnrfifHhDYfgasaacH8akY=wiFfYdH8Gipec8Eeeu0xXdbba9frFj0=OqFfea0dXdd9vqai=hGuQ8kuc9pgc9s8qqaq=dirpe0xb9q8qiLsFr0=vr0=vr0dc8meaabaqaciaacaGaaeqabaqabeGadaaakeaacqWGdbWqdaqhaaWcbaGaeGOmaidabaGaeGioaGdaaOGaeyypa0ZaaSaaaeaacqaI4aaocqGGHaqiaeaacqaIYaGmcqGGHaqicqGGOaakcqaI4aaocqGHsislcqaIYaGmcqGGPaqkcqGGHaqiaaGaeyypa0JaeGOmaiJaeGioaGdaaa@3CCB@. In addition, the eight combinations with the same power are added. The eight powers and the 36 combinations are tested consecutively. For the first-degree FP, the differences in deviance of each model from the linear one are calculated and compared with the chi-square distribution, with one degree of freedom at α = 0.05. For the second-degree FP, the differences in deviance of each model from the best fitting first-degree FP are calculated and compared with the chi-square distribution with two degrees of freedom. The model with the largest significant deviance difference compared to the best first-degree FP is selected as the best second-degree FP. The second-degree FP is therefore implicitly a better fit than either the linear model or the first-degree FP. Using the same example as in the first-degree FP; the second-degree FP model is defined mathematically as follows:

log*it*(*π*_*ib*_) = *α *+ *β*_1_H_1_(*X*_*i*_) + *β*_1_H_1_(*X*_*i*_) + *γW*_*b*_

..where, H_1_(X) and H_2_(X) are the respective functions to which co-variable X is transformed.

The rate ratio for the second-degree FP is given by the formula:

*RR *= exp (*β*_1 _* (H_1_(*X*_1_) - *H*_1_(*X*_0_)) + *β*_2 _* (H_2_(*X*_1_) - *H*_2_(*X*_0_)))

All the models were run and fit using STATA ^® ^software [23]. The output of each model (coefficients and transformed variables) was used to calculate rate ratios (RR), only including variables with a significant effect on the risk of clinical malaria. These are mean temperature, rainfall, and relative humidity. Other variables, including minimum and maximum temperature, were removed from the final model by an in-built backward elimination procedure in the FP algorithm. For each variable, reference points **(*x***_0_**) **were defined. These are mean temperature = 27°C, the value at which clinical malaria risk peaked; rainfall = 164 mm, the highest value observed; and relative humidity = 60%, the minimum value required for malaria vector survival.

## Results

### Study population

#### Characteristics

Table 1 provides a summary of the study population characteristics at the beginning of the observation period. In total, 676 children distributed in 350 households took part in the study. The average number of children per household was 1.9. Overall, females were more (52.8%) than males, but the sex distribution was not significantly different across the sites (p = 0.420). Similarly, the age distribution did not differ between sites (p = 0.938).

**Table 1:** Study population characteristics at the beginning of the study

			**Sites**						
				
	**All**	**(%)**	**Cissé**	**(%)**	**Goni**	**(%)**	**Nouna**	**(%)**	*χ^2 ^***test***
**n**	**676**		**171**		**240**		**265**		
**Household**	350		74		125		151		
**Children per household**	1.9		2.3		1.9		1.8		
**Gender**									*p value *= 0.420
Female	357	(52.8)	103	(60.2)	116	(48.3)	138	(52.1)	
Male	319	(47.2)	68	(39.8)	124	(51.7)	127	(47.9)	
**Age in months**									*p value *= 0.929
< 12	65	(09.6)	14	(08.2)	19	(07.9)	32	(12.1)	
12–23	157	(23.2)	39	(22.8)	57	(23.8)	61	(23.0)	
24–35	159	(23.5)	36	(21.1)	59	(24.6)	64	(24.2)	
36–47	161	(23.8)	40	(23.4)	58	(24.2)	63	(23.8)	
48–59	134	(19.8)	42	(24.6)	47	(19.6)	45	(17.0)	

* Mantel-Haenszel chi-square analysis used to detect inter- site differences.

#### Follow up status

During the one-year follow up, 20 children (3.0% of 676) left the cohort, either because of death (11) or migration out of the study sites (9). Although 52 home visits were planned for each child, on average, each child was seen for 45.4 times, because children were not always present at each visit. In total 594.9 person-years (PYs) were observed.

#### Meteorological conditions

**Figure 3:**
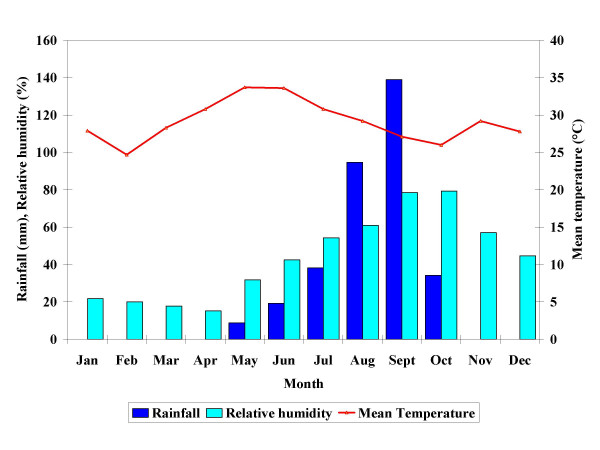
Monthly rainfall, mean temperature and relative humidity for Cissé.

**Figure 4:**
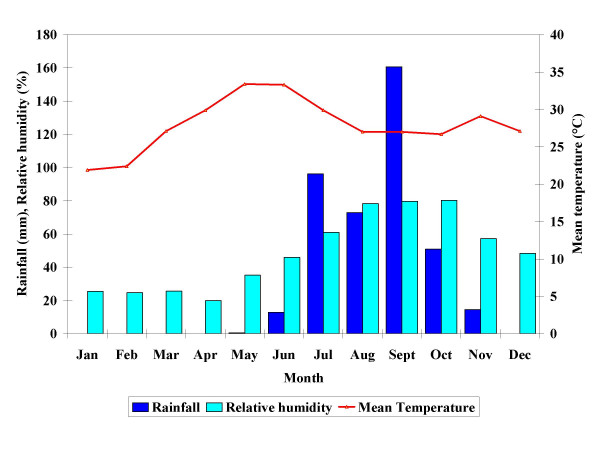
Monthly rainfall, mean temperature and relative humidity for Goni.

**Figure 5:**
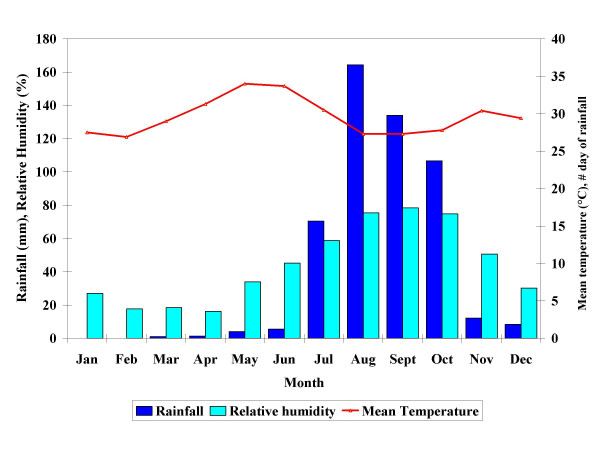
Monthly rainfall, mean temperature and relative humidity for Nouna.

The three sites presented a similar pattern of meteorological conditions (Figure 3, 4, 5). Rainfall was observed in all sites from May to November; the highest amounts were observed in August, September, and October. The total amount of rainfall was higher in Nouna (508. 3 mm in 68 days) than in Cissé (334 mm in 49 days) or Goni (408.5 mm in 54 days). The pattern of relative humidity followed the one of rainfall. At all sites, relative humidity was low during the first six months of the year, and then increased with the onset of the rains. The average relative humidity over the study period was higher in Goni (48.5%; range 10.2–89.8%; SD: 22.8) compared to Cissé (43.7%; range: 9.5–89.6%; SD: 23.1) and Nouna (44.0%; range: 10.4–89.4%; SD: 23.8). The mean temperature was similar across all sites. With the onset of the rains, temperatures decreased to 26.6°C in Cissé and Nouna and 26.5°C in Goni between August and September. From September on, the temperature rose again; and then started to decrease in December, the coldest month. The average mean temperature for the whole period was lower in Goni, though with large variance (27.9°C; range: 20.8–35.9; SD: 3.8) compared to Cissé (29.1°C; range 22.8–35.9; SD: 3.1) and Nouna (29.6°C; range 20.6–35.4; SD 2.9).

The mean daily temperature variation in a month, expressed by standard deviation, was high in March for all sites (Cissé: 3.1; Goni: 2.9; and Nouna: 2.9). From March onwards, at all sites, this variation decreased in all sites to achieve its smallest value in December, which was 0.7 and 1.1 in Goni and Nouna, respectively. An unusual small variation was observed in Cissé in October (0.1).

#### Clinical malaria incidence

Over the study period, 1274 episodes of fever were observed; thus, an incidence of 2.1 episodes per PY (1274 episodes/594.9 PYs), which was similar in all sites (Cissé = 2.1, Goni = 2.3, and Nouna = 2.0). Out of 1274 fever episodes, 635 had parasiteamia and were therefore considered as cases of clinical malaria, giving an incidence of 1.1 episodes per PY. Nouna had the lowest incidence of 0.8 per PY. In Cissé and Goni, the incidence was 1.2 and 1.3 per PY, respectively.

Table 2 presents the monthly distribution of clinical malaria incidence which varied strongly between months. The lowest incidence rates per 1000 person months (PM) were in May and June (Cissé: 8.1, 7.7; Goni: 37.7, 35.3 and Nouna: 26.7, 15.1, respectively) in all sites. The overall monthly incidences for May and June were 26.2 and 20.8 per 1000 PM, respectively. In contrast, the highest incidence was observed in different months; in Cissé, incidence peaked in August (326.5 per 1000 PM) versus September for Goni (331.3 per 1000 PM) and Nouna (130.6 per 1000 PM) (Table 2).

**Table 2:** Monthly under-five incidence of clinical malaria in the three study sites

	**Sites**											
				
	**Cissé**			**Goni**			**Nouna**			**All**		
**Month**	**PM**	**CE**	**IR**	**PM**	**CE**	**IR**	**PM**	**CE**	**IR**	**PM**	**CE**	**IR**

December 2003	149.8	29	193.6	221.4	27	122.0	185.0	20	108.1	556.2	76	136.6
January 2004	139.5	6	43.0	215.8	8	37.1	208.4	7	33.6	563.7	21	37.3
February 2004	139.8	19	135.9	213.3	8	37.5	211.6	12	56.7	564.7	39	69.1
March 2004	145.9	18	123.4	210.8	18	85.4	212.6	11	51.7	569.3	47	82.6
April 2004	139.3	2	14.4	205.8	12	58.3	210.5	26	123.5	555.6	40	72.0
May 2004	123.2	1	8.1	185.6	7	37.7	187.3	5	26.7	496.1	13	26.2
June 2004	130.7	1	7.7	198.1	7	35.3	198.3	3	15.1	527.1	11	20.9
July 2004	142.6	2	14.0	209.5	23	109.8	199.5	7	35.1	551.6	32	58.0
August 2004	131.7	43	326.5	197.5	53	268.4	197.7	20	101.2	526.9	116	220.2
September 2004	125.6	25	199.0	181.1	60	331.3	176.1	23	130.6	482.8	108	223.7
October 2004	140.9	18	127.8	207.9	41	197.2	208.6	26	124.6	557.4	85	152.5
November 2004	139.8	12	85.8	207.2	23	111.0	208.1	12	57.7	555.1	47	84.7

**Total**	**150.9**	**176**	**1166.3**	**224.4**	**287**	**1279.0**	**219.6**	**152**	**692.2**	**594.9**	**635**	**1067.4**

PM: Person months, CE: Clinical malaria cases, IR: Incidence Rate per1000 PM (per PY for the total)

### Effect of meteorological parameters on incidence of clinical malaria episodes

#### Fractional polynomial transformation algorithm

Table 3 shows the transformation functions for each variable. The relationships between meteorological parameters and clinical malaria incidence were not linear since none of the functions had the power of +1. Minimum and maximum temperatures, which were included in the initial model, were removed by backward elimination. Only the interaction terms of 'temperature and humidity', and 'temperature and rainfall' had a linear relationship with first-degree transformation. The best fit for mean temperature, rainfall and relative humidity alone were obtained with second-degree transformations. For the interaction term 'rainfall and humidity', the first-degree transformation with a power of -1 yielded the best fit.

**Table 3:** Meteorological variables, their transformation powers and functional forms used in deriving estimates for the association with clinical malaria risk

**Variables (degree)**	**Variables (power)**	**Transformation functions**
Average temperature (Second degree)	Tmean_1 (-2)	(Tmean10)−2−0.1201 MathType@MTEF@5@5@+=feaafiart1ev1aaatCvAUfKttLearuWrP9MDH5MBPbIqV92AaeXatLxBI9gBaebbnrfifHhDYfgasaacH8akY=wiFfYdH8Gipec8Eeeu0xXdbba9frFj0=OqFfea0dXdd9vqai=hGuQ8kuc9pgc9s8qqaq=dirpe0xb9q8qiLsFr0=vr0=vr0dc8meaabaqaciaacaGaaeqabaqabeGadaaakeaadaqadaqaamaalaaabaGaemivaqLaemyBa0MaemyzauMaemyyaeMaemOBa4gabaGaeGymaeJaeGimaadaaaGaayjkaiaawMcaamaaCaaaleqabaGaeyOeI0IaeGOmaidaaOGaeyOeI0IaeGimaaJaeiOla4IaeGymaeJaeGOmaiJaeGimaaJaeGymaedaaa@3F4F@
	Tmean_2 (0.5)	(Tmean10)0.5−0.1699 MathType@MTEF@5@5@+=feaafiart1ev1aaatCvAUfKttLearuWrP9MDH5MBPbIqV92AaeXatLxBI9gBaebbnrfifHhDYfgasaacH8akY=wiFfYdH8Gipec8Eeeu0xXdbba9frFj0=OqFfea0dXdd9vqai=hGuQ8kuc9pgc9s8qqaq=dirpe0xb9q8qiLsFr0=vr0=vr0dc8meaabaqaciaacaGaaeqabaqabeGadaaakeaadaqadaqaamaalaaabaGaemivaqLaemyBa0MaemyzauMaemyyaeMaemOBa4gabaGaeGymaeJaeGimaadaaaGaayjkaiaawMcaamaaCaaaleqabaGaeGimaaJaeiOla4IaeGynaudaaOGaeyOeI0IaeGimaaJaeiOla4IaeGymaeJaeGOnayJaeGyoaKJaeGyoaKdaaa@4064@
Rainfall (Second degree)	Pmm_1 (2)	(Pmm+0.3000100)2−0.1261 MathType@MTEF@5@5@+=feaafiart1ev1aaatCvAUfKttLearuWrP9MDH5MBPbIqV92AaeXatLxBI9gBaebbnrfifHhDYfgasaacH8akY=wiFfYdH8Gipec8Eeeu0xXdbba9frFj0=OqFfea0dXdd9vqai=hGuQ8kuc9pgc9s8qqaq=dirpe0xb9q8qiLsFr0=vr0=vr0dc8meaabaqaciaacaGaaeqabaqabeGadaaakeaadaqadaqaamaalaaabaGaemiuaaLaemyBa0MaemyBa0Maey4kaSIaeGimaaJaeiOla4IaeG4mamJaeGimaaJaeGimaaJaeGimaadabaGaeGymaeJaeGimaaJaeGimaadaaaGaayjkaiaawMcaamaaCaaaleqabaGaeGOmaidaaOGaeyOeI0IaeGimaaJaeiOla4IaeGymaeJaeGOmaiJaeGOnayJaeGymaedaaa@4326@
	Pmm_2 (2)	(Pmm+0.3000100)2*ln⁡(Pmm+0.3000100)+0.1306 MathType@MTEF@5@5@+=feaafiart1ev1aaatCvAUfKttLearuWrP9MDH5MBPbIqV92AaeXatLxBI9gBaebbnrfifHhDYfgasaacH8akY=wiFfYdH8Gipec8Eeeu0xXdbba9frFj0=OqFfea0dXdd9vqai=hGuQ8kuc9pgc9s8qqaq=dirpe0xb9q8qiLsFr0=vr0=vr0dc8meaabaqaciaacaGaaeqabaqabeGadaaakeaadaqadaqaamaalaaabaGaemiuaaLaemyBa0MaemyBa0Maey4kaSIaeGimaaJaeiOla4IaeG4mamJaeGimaaJaeGimaaJaeGimaadabaGaeGymaeJaeGimaaJaeGimaadaaaGaayjkaiaawMcaamaaCaaaleqabaGaeGOmaidaaOGaeiOkaOIagiiBaWMaeiOBa42aaeWaaeaadaWcaaqaaiabdcfaqjabd2gaTjabd2gaTjabgUcaRiabicdaWiabc6caUiabiodaZiabicdaWiabicdaWiabicdaWaqaaiabigdaXiabicdaWiabicdaWaaaaiaawIcacaGLPaaacqGHRaWkcqaIWaamcqGGUaGlcqaIXaqmcqaIZaWmcqaIWaamcqaI2aGnaaa@5583@
Relative humidity (Second degree)	RH_1 (-1)	(RH10)−1*ln⁡(RH10)−0.3332 MathType@MTEF@5@5@+=feaafiart1ev1aaatCvAUfKttLearuWrP9MDH5MBPbIqV92AaeXatLxBI9gBaebbnrfifHhDYfgasaacH8akY=wiFfYdH8Gipec8Eeeu0xXdbba9frFj0=OqFfea0dXdd9vqai=hGuQ8kuc9pgc9s8qqaq=dirpe0xb9q8qiLsFr0=vr0=vr0dc8meaabaqaciaacaGaaeqabaqabeGadaaakeaadaqadaqaamaalaaabaGaemOuaiLaemisaGeabaGaeGymaeJaeGimaadaaaGaayjkaiaawMcaamaaCaaaleqabaGaeyOeI0IaeGymaedaaOGaeiOkaOIagiiBaWMaeiOBa42aaeWaaeaadaWcaaqaaiabdkfasjabdIeaibqaaiabigdaXiabicdaWaaaaiaawIcacaGLPaaacqGHsislcqaIWaamcqGGUaGlcqaIZaWmcqaIZaWmcqaIZaWmcqaIYaGmaaa@4469@
	RH_2 (-1)	(RH10)−1*ln⁡(RH10)−0.3332 MathType@MTEF@5@5@+=feaafiart1ev1aaatCvAUfKttLearuWrP9MDH5MBPbIqV92AaeXatLxBI9gBaebbnrfifHhDYfgasaacH8akY=wiFfYdH8Gipec8Eeeu0xXdbba9frFj0=OqFfea0dXdd9vqai=hGuQ8kuc9pgc9s8qqaq=dirpe0xb9q8qiLsFr0=vr0=vr0dc8meaabaqaciaacaGaaeqabaqabeGadaaakeaadaqadaqaamaalaaabaGaemOuaiLaemisaGeabaGaeGymaeJaeGimaadaaaGaayjkaiaawMcaamaaCaaaleqabaGaeyOeI0IaeGymaedaaOGaeiOkaOIagiiBaWMaeiOBa42aaeWaaeaadaWcaaqaaiabdkfasjabdIeaibqaaiabigdaXiabicdaWaaaaiaawIcacaGLPaaacqGHsislcqaIWaamcqGGUaGlcqaIZaWmcqaIZaWmcqaIZaWmcqaIYaGmaaa@4469@
Rainfall and humidity (first degree)	PmmRH (-1)	(PmmRH+2.4699974110000)−1−3.971 MathType@MTEF@5@5@+=feaafiart1ev1aaatCvAUfKttLearuWrP9MDH5MBPbIqV92AaeXatLxBI9gBaebbnrfifHhDYfgasaacH8akY=wiFfYdH8Gipec8Eeeu0xXdbba9frFj0=OqFfea0dXdd9vqai=hGuQ8kuc9pgc9s8qqaq=dirpe0xb9q8qiLsFr0=vr0=vr0dc8meaabaqaciaacaGaaeqabaqabeGadaaakeaadaqadaqaamaalaaabaGaemiuaaLaemyBa0MaemyBa0MaemOuaiLaemisaGKaey4kaSIaeGOmaiJaeiOla4IaeGinaqJaeGOnayJaeGyoaKJaeGyoaKJaeGyoaKJaeG4naCJaeGinaqJaeGymaedabaGaeGymaeJaeGimaaJaeGimaaJaeGimaaJaeGimaadaaaGaayjkaiaawMcaamaaCaaaleqabaGaeyOeI0IaeGymaedaaOGaeyOeI0IaeG4mamJaeiOla4IaeGyoaKJaeG4naCJaeGymaedaaa@4B71@
Temperature and humidity (first degree)	TRH (1)	*TRH *- 1305
Temperature and rainfall (first degree)	TPmm (1)	*TPmm *- 989.7

#### Model estimates of coefficients and confidence limits

Although other variables were included in the model for adjustment purposes, their estimates are not shown in Table 4, because the focus was on meteorological parameters. For each power of transformation, an estimate is given. All variables, individually or combined, had a significant effect on clinical malaria rates. The highest effect was observed with mean temperature. The combined effects of rainfall-humidity, temperature-humidity, and temperature-rainfall, although significant, were small.

**Table 4:** Model estimates and confidence intervals for the meteorological variables

**Variables**	***β*estimates**	**95% CI**
Tmean_1	-86.9789	(-113.4057 ; -60.5521)
Tmean_2	-29.7873	(-38.7457 ; -20.8288)
Pmm_1	3.7666	(1.5279 ; 6.0053)
Pmm_2	-3.4380	(-5.3612 ; -1.5147)
RH_1	-8.9203	(-14.807 ; -3.0337)
RH_2	-23.2151	(-36.7151 ; -9.7152)
PmmRH	-0.0003	(-0.0004 ; -0.0002)
TRH	-0.0035	(-0.0058 ; -0.0012)
TPmm	-0.0012	(-0.0021 ; -0.0004)

Intercept	-3.8180	(-4.18581 ; -3.4502)

Log pseudo-likelihood = -2920.2374, Wald chi square = 282, Deviance: 5840.466

#### Rate Ratio of clinical malaria

**Figure 6:**
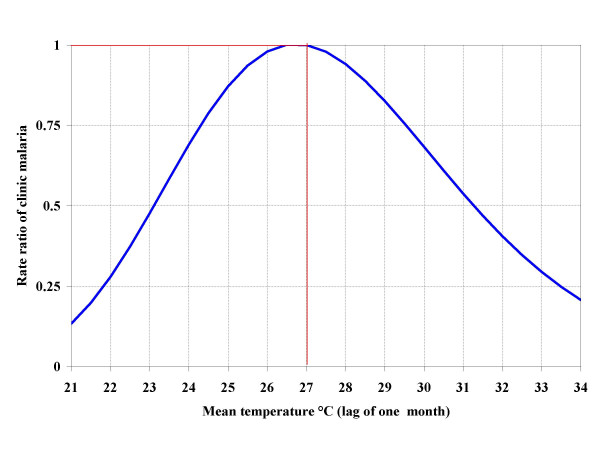
Effect of mean temperature on clinical malaria risk (RR = rate ratio) among study children. Horizontal and vertical red lines indicate the reference point (RR = 1; T = 27°C).

**Figure 7:**
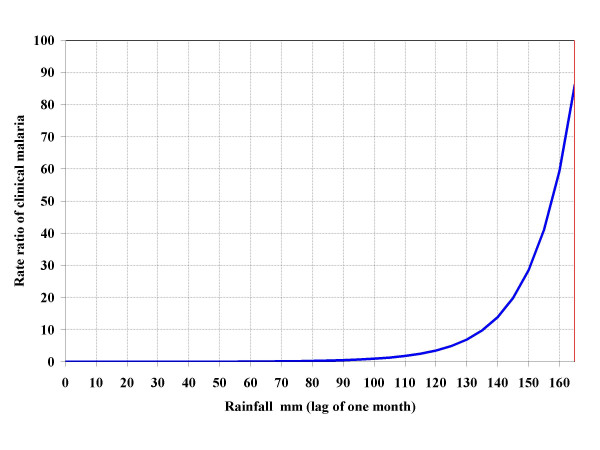
Effect of total rainfall on clinical malaria risk (RR = rate ratio) among study children. Horizontal and vertical red lines indicate the reference point (RR = 1, RH = 60%).

**Figure 8:**
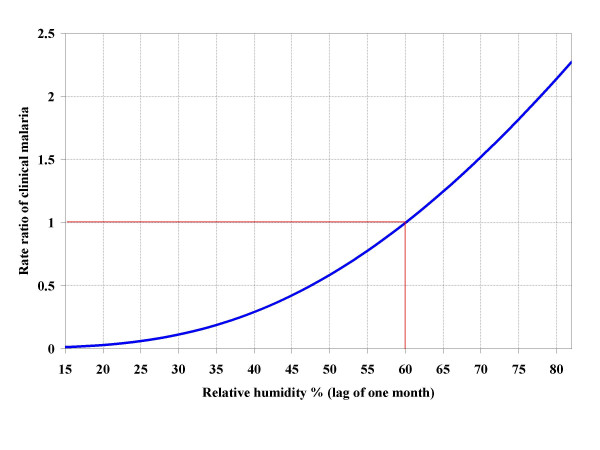
Effect of relative humidity on clinical malaria risk (RR = rate ratio) among study children. Horizontal and vertical red lines indicate the reference point (RR = 1, RH = 60%).

The effects of meteorological parameters on clinical malaria expressed in rate ratios (RR) are presented in Figure 6, 7 and 8. For each parameter, vertical and horizontal lines show the reference point.

Mean temperature ranged from 21°C to 34°C. The relationship between mean temperature and clinical malaria incidence displayed a bell-shape curve (Figure 6). The risk of clinical malaria increased with an increase in mean temperature up to 27°C. At 23°C, the risk for clinical malaria was 53% of the risk at 27°C. Temperatures above 27°C led to a significant decrease in clinical malaria risk. The risk was therefore least at the lower and higher extremes of the temperature range.

The effect of rainfall on clinical malaria risk was observed only for values above 100 mm (Figure 7). Below that level, the risk reduction compared to 164 mm was almost 100%. There was no difference in risk between the rainfall levels of 60 mm and 90-mm. Above 100 mm, the risk increased significantly for each increase of 10 mm of rain. For example, the RR at 150 mm was 0.3 compared to 0.7 at 160 mm.

Relative humidity observed in the field ranged between 15% and 80%. The clinical malaria risk increased with an increase in relative humidity, but not linearly (Figure 8). Below 60% humidity, there was low risk. For example at 55% relative humidity, the risk was 25% lower than at 60% relative humidity. The risk of clinical malaria increased exponentially at relative humidity exceeding 60%.

## Discussion

The individual effects of temperature, rainfall, and relative humidity of the preceding month on clinical malaria risk among children under age five were explored. The results confirmed that all meteorological factors are predictors for malaria risk. However, mean temperature was the best predictor.

Mean temperature alone was a strong predictor of clinical malaria rates among the children in our sample. Its relationship with clinical malaria was bell-shaped such that the risk was lowest at low and high temperatures. The infection risk was highest at temperatures around 27°C. There is no direct biological link between human infection and ambient temperature. However, the temperature-dependence of the *Anopheles gambiae *(the primary malaria vector in this region) life cycle could explain these results. Indeed, the sporogonic cycle (the development of the parasite within the vector) is shorter (9–10 days) at 28°C and longer (about 100 days) at temperatures below 20°C [1,24]. Therefore, a short incubation period allows the vector to live long enough to become infectious. The lifespan of the *Anopheles gambiae *is about 21 days. At high temperatures, vector survival decreases and temperatures of 40°C will result in zero vector survival. Craig and colleagues [4] reported that the proportion of vectors surviving the incubation period is high at temperatures between 28°C and 32°C. This explains the high-risk of clinical malaria observed in this range of temperature. In addition, temperatures above 27°C increase the feeding frequency of the vector to every two days, as the blood digestion rate increases. This results in more frequent vector-human contact. These findings agree with those from several other studies [25,26,7,9].

Cumulative rainfall from the previous month had a positive effect on clinical malaria rates as well. Several other studies reported similar results [26,6,9,10]. The relationship was 'J-shaped', suggesting that the relationship is non-linear, contrary to the results reported by Teklehiemanot and colleagues [9]. In addition, a minimum amount of rainfall is necessary for transmission to take place. In our study, a minimum of 100 mm was necessary before any case of clinical malaria could be observed. This could be explained by the hot and dry climate of the study area leading to high evaporation and water infiltration into the soil. Small amounts of rainfall will evaporate or infiltrate over a shorter time. Conversely, with higher amounts of rainfall, some water remains long enough for the vector to complete its development cycle. At higher amounts of rainfall, further increases may have little effect on clinical malaria risk, suggesting that a saturation level exists, as reported by Teklehiemanot and colleagues [9]. However, because of the relatively low amount of maximum rainfall (160 mm), the model could not capture this saturation phenomenon. As with temperature, the effect of rainfall is not directly related to human infection; but rainfall influences vector population abundance by providing open surface water for breeding. A positive effect of rainfall on malaria transmission has been reported widely in the literature, even though some studies have shown a converse or negligible effect [27-30,7]. This might be explained by the overwhelming effect of temperature. For rainfall to have a positive effect on malaria incidence, the temperature must be warm enough to support mosquito and parasite development [31]. This explains the low rate of malaria transmission in highlands, where rainfall is abundant, but temperatures are relatively low (below 20°C).

The individual effect of relative humidity on risk of clinical malaria, compared to temperature and rainfall, has been reported less often in the literature. Mean relative humidity of the previous month had a positive effect on malaria incidence. The effect was greater than that of rainfall. This is in line with the findings of Bi and colleagues [32]. To account for the strong correlation between rainfall and relative humidity an interaction term was included in the model. Thus, the estimate represents the individual effect of relative humidity on clinical malaria risk. The relative humidity observed ranged from 15% to 80%, with an average of 50%, mainly driven by rainfall and temperature. At a relative humidity below 60%, there was a decline in the risk of clinical malaria. This could be explained by a decrease in vector lifespan under these conditions, therefore, reducing the proportion of vector survival [33]. In contrast, at relative humidity above 60%, the infection rate increases substantially as humidity increases. The risk at 80% humidity is twice the risk at 60%. Again, this can be explained by improved vector survival.

The study was performed only for one year; thus, it cannot claim to be representative of all years. In fact, rainfall and humidity were both below the average of the last 10 years (data from the national meteorological station), but average temperatures were similar. Clinical data from several years (at least three years) of observation would have been ideal; but this was not feasible, given the costs involved in collecting the malaria infection data. In addition, the reported malaria incidence was not significantly different from that reported in a study conducted in the preceding year. Without a catastrophic event (like flooding or drought), the effect of meteorological variables on malaria infection should not change much from one year to another.

In such a semi-immune population, malaria may be transient. Therefore, weekly visits may not be sensitive enough to capture all the episodes. Infection acquired between visits could have been treated and cured and therefore missed. Indeed, Snow and colleagues reported a sensitivity of 74% of weekly visits in capturing clinical malaria episodes compared to daily visits [34]. In this setting, we expect a slightly higher sensitivity than the 74% because the most commonly used drug at the time was chloroquine which was reported to have a treatment failure of 36% [35]. Also, unless the child was severely ill, parents preferred to wait for the next visit of the interviewer because treatment of study participants was free. It should also be noted that Snow and colleagues did not find statistically significant differences in the risk of detecting malaria episodes when comparing daily visits to weekly ones [34]. Considering the cost and logistics involved in daily visits, the more cost-effective alternative was the weekly visits. The probable underestimation of the clinical malaria episodes notwithstanding, we do not expect the nature of the relationships with meteorological parameters to differ greatly from our findings.

## Conclusion

The findings of this study confirm that meteorological conditions have a significant influence on clinical malaria rates among children under-five years old. Although several individual meteorological parameters have an impact on clinical malaria incidence, mean temperature is the best predictor and the main driving force, at least in the region. This is also observed in other regions. For example in the highlands region the preventing factor for malaria transmission is the low temperature because of the high altitude. Changes in ecological settings because of climate change are to be expected in this sub-Saharan region, especially the rain patterns. These changes may modify local mosquito microhabitats and affect transmission widely. A health information system including systematic monitoring of temperature and rainfall could yield an early warning system to support malaria control efforts at district level.

## Competing interests

The author(s) declare that they have no competing interests.

## Authors' contributions

YY designed and implemented the study. He performed the statistical analysis and drafted the manuscript. VL took part in statistical analysis, writing of the manuscript and interpretation of the findings. SS supervised the fieldwork and contributed in the writing. RS participated in the design, implementation, and writing of the manuscript. All authors read and approved the final manuscript

## Pre-publication history

The pre-publication history for this paper can be accessed here:


